# Synergistic D‐Amino Acids Based Antimicrobial Cocktails Formulated via High‐Throughput Screening and Machine Learning

**DOI:** 10.1002/advs.202307173

**Published:** 2023-12-21

**Authors:** Jingzhi Yang, Yami Ran, Shaopeng Liu, Chenhao Ren, Yuntian Lou, Pengfei Ju, Guoliang Li, Xiaogang Li, Dawei Zhang

**Affiliations:** ^1^ Beijing Advanced Innovation Center for Materials Genome Engineering Institute for Advanced Materials and Technology University of Science and Technology Beijing Beijing 100083 China; ^2^ National Materials Corrosion and Protection Data Center University of Science and Technology Beijing Beijing 100083 China; ^3^ BRI Southeast Asia Network for Corrosion and Protection Shunde Graduate School of University of Science and Technology Beijing Foshan 528000 China; ^4^ Shanghai Aerospace Equipment Manufacturer Shanghai 200245 China; ^5^ College of Materials Science and Engineering Beijing University of Chemical Technology Beijing 100029 China

**Keywords:** antimicrobial resistance, D‐amino acids, high‐throughput screening, machine learning

## Abstract

Antimicrobial resistance (AMR) from pathogenic bacterial biofilms has become a global health issue while developing novel antimicrobials is inefficient and costly. Combining existing multiple drugs with enhanced efficacy and/or reduced toxicity may be a promising approach to treat AMR. D‐amino acids mixtures coupled with antibiotics can provide new therapies for drug‐resistance infection with reduced toxicity by lower drug dosage requirements. However, iterative trial‐and‐error experiments are not tenable to prioritize credible drug formulations, owing to the extremely large number of possible combinations. Herein, a new avenue is provide to accelerate the exploration of desirable antimicrobial formulations via high‐throughput screening and machine learning optimization. Such an intelligent method can navigate the large search space and rapidly identify the D‐amino acid mixtures with the highest anti‐biofilm efficiency and also the synergisms between D‐amino acid mixtures and antibiotics. The optimized drug cocktails exhibit high antimicrobial efficacy while remaining non‐toxic, which is demonstrated not only from in vitro assessments but also the first in vivo study using a lung infection mouse model.

## Graphical abstract

This study proposes an advanced and efficient methodology that combines a high‐throughput experimental platform and machine learning optimization to effectively design potent antimicrobial cocktails out of the combinations of virtually all D‐amino acids and common antibiotics. The discovered gentamicin/D‐mix cocktail therapy presents high treatment efficiency in vivo with almost no toxicity, providing important validation towards future clinical uses.

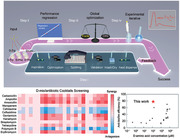

## 1 Introduction

Microbial infection has been identified as a major health threat due to the presence of antimicrobial resistance (AMR), which refers to the resistance of bacteria to the effects of antimicrobial agents.^[^ ^1^ ^]^ It is estimated that drug‐resistant microbial infection may result in 10 million deaths each year globally, and an enormous cost of 100 trillion USD by 2050.^[^ ^2^, ^3^ ^]^ The origin of AMR is primarily related to the survival strategies of bacteria. Bacterial cells tend to colonize on surfaces and form biofilms, wherein they are bound together by self‐produced extracellular polymeric substances (EPS).^[^ ^4^, ^5^ ^]^ Biofilms provide essential nutrients for bacterial growth and protect bacteria from environmental disturbances such as immune responses and antimicrobial agents.^[^ ^6^, ^7^ ^]^ Compared with planktonic bacterial cells, bacteria encapsulated in biofilms exhibit stronger resistance to antimicrobials. The obstinate defense of biofilm makes pathogens demand more powerful antimicrobial treatments to eradicate microbial infection. Thus, material surfaces with enhanced anti‐biofilm properties are particularly interesting in antimicrobial research.^[^ ^8^, ^9^ ^]^

D‐amino acids have been recently applied as biocompatible anti‐biofilm agents. Bacteria show minimal rejection of exogenous D‐amino acids, which can be naturally found in both eukaryotic and bacterial cells^[^ ^10^, ^11^, ^12^ ^]^ and play very important roles in bacterial homeostasis.^[^ ^13^, ^14^ ^]^ The anti‐biofilm effect of D‐amino acids is attributed to their ability to disassemble protein fibers and inhibit the accumulation of the protein component in the bacterial biofilm matrix.^[^ ^15^ ^]^ Such effect was first found for *S. aureus* ^[^ ^16^ ^]^ and *P. aeruginosa* ^[^ ^11^ ^]^ and subsequently demonstrated in the studies of a variety of bacteria treated with all 18 exogenous D‐amino acids alone or in combination. The results from these studies indicated that the cells exposed to D‐amino acid mixtures were more significantly impaired in biofilm formation than D‐amino acids alone. For example, Hochbaum et al. showed a mixture of equimolar D‐tyrosine (D‐Tyr), D‐proline (D‐Pro), and D‐phenylalanine (D‐Phe) was more effective in inhibiting the biofilm formation of *S. aureus* SC01 than any of the individual amino acids.^[^ ^16^ ^]^ Sanchez et al. reported that the combination of D‐Tyr, D‐leucine (D‐Leu), D‐methionine (D‐Met), and D‐tryptophan (D‐Trp) caused a more significant reduction in cell biomass.^[^ ^17^ ^]^ D‐amino acids may also be used as enhancers for antibiotics which could not only significantly improve their efficacy but also minimize the toxicity.^[^ ^18^, ^19^, ^20^, ^21^ ^]^ Wu et al. indicated that D‐Tyr supplementation weakened the resistance of *P. aeruginosa* to amikacin, mitigating the toxicity by preventing the overuse of amikacin.^[^ ^18^ ^]^ Warraich et al. confirmed that D‐amino acids targeting matrix components increased the efficacy of ciprofloxacin and were able to effectively treat AMR.^[^ ^21^ ^]^ Although the efficacy of D‐amino acids as antimicrobial treatment has been well demonstrated by in vitro tests, few studies were found reporting the in vivo performance of D‐amino acids. Recently, Iwata et al. showed that D‐Ser could inhibit murine peritonitis caused by catheter infection.^[^ ^22^ ^]^ Despite the desirable properties of D‐amino acids, identification of high‐efficiency D‐amino acids and their mixtures are mostly achieved based on trial‐and‐error experiments, which are formidable tasks considering the number of the combinations of various D‐amino acids. A more efficient and economical method is needed to accelerate the discovery of the D‐amino acids mixed in a way to achieve optimal anti‐biofilm properties.

Machine learning is an active learning method that starts with “a small dataset” and augments the experimental results into the training data on‐the‐fly to accelerate the process of finding the potential target solution.^[^ ^23^, ^24^ ^]^ In recent years, machine learning algorithms have been extensively applied in a variety of fields such as biomedicine and materials science,^[^ ^25^, ^26^, ^27^, ^28^, ^29^, ^30^ ^]^ and exhibited great potential for design and optimized screening of therapeutics. For example, Stokes et al. employed a neural network model to predict the antimicrobial activity of new compounds with unknown molecular structures. They readily discovered two antimicrobial compounds with potent broad‐spectrum activity out of the total 107 million molecules, which also could overcome the AMR to *E. coli*.^[^ ^31^ ^]^ Zoffmann et al. adopted a machine learning‐powered data analysis to identify novel antibiotics based on compound fingerprint similarity.^[^ ^32^ ^]^ In cancer therapies, machine learning models have been utilized to predict the drug synergy score.^[^ ^29^ ^]^

In this study, we proposed an advanced approach based on high‐throughput experiments and machine learning optimization to rapidly and systematically identify the high‐efficiency AMR treatments in the vast chemical space of D‐amino acids and antibiotics. A high‐throughput platform based on a non‐contact droplet microarray printer was employed to build the input dataset for machine learning given that the experimental data from predecessors are scarce. An adaptive procedure combining a machine learning regression model and an efficient global optimization (EGO) algorithm was adopted to search for D‐amino acid mixtures with improving anti‐biofilm properties. This approach provided a robust and guided antimicrobial formulation design strategy to navigate the potential space (≈10626 combinations) and select the next experimental candidate via expected improvement (EI). Taking full consideration of the trade‐off between exploitation and exploration, this approach is expected to avoid the local minima of the search space. We experimentally characterized the feedback of the EGO, i.e., the D‐amino acid mixtures that showed more effective anti‐biofilm performance than any of the original input datasets obtained from high‐throughput screening. Moreover, we identified a large number of high‐dimensional combinations of D‐amino acids and antibiotics by the high‐throughput experimental platform. The lowest effective concentrations (LEC) of various antibiotics, in vitro antimicrobial properties and toxicity of the antimicrobial cocktails were sequentially screened to evaluate the synergy between the D‐amino acids and the antibiotics. The in vivo antimicrobial efficacy of the optimized cocktails therapy was demonstrated in *P. aeruginosa* lung infection mouse models.

## 2 Results

### 2.1 Machine Learning Design Loop for D‐Amino Acid Mixtures Optimization

**Figure 1:**
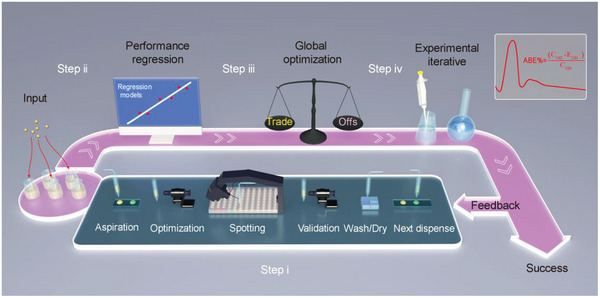
A) D‐amino acid mixture design strategy for targeted properties optimization based on a high‐throughput screening and machine learning approach.

**Figure** **1** illustrated the design strategy for D‐amino acid mixtures with an optimized anti‐biofilm performance by the high‐throughput screening and machine learning approach. The machine learning design loop included four main steps: i) input dataset construction, ii) model selection, iii) global optimization and iv) experimental iterative feedback. We used a high‐throughput experimental platform (Figure S1, Supporting Information) to screen the biofilm formation inhibitory efficiency of D‐amino acid mixtures which were combined at most in pentabasic form (see section 2.2). A machine learning classification model that used the input dataset to learn the feature–property relationship was built and applied to extrapolate the chemical space of unexplored D‐amino acids compositions (of which the anti‐biofilm performance has not been measured before) (see section 2.3). An active learning method based on the EGO algorithm was used to explore regions where the model was less accurate (the spaces with high EI values). Subsequently, the EGO guided experimental data was added to the original input dataset allowing the iterative improvement of the regression model (see section 2.4). After several rounds of optimization and iteration, the design loop ended when the D‐amino acid mixtures with optimal anti‐biofilm performance against *P. aeruginosa* were found (see section 2.5).

### 2.2 Input Dataset Construction

The input dataset construction was achieved by high‐throughput screening with a non‐contact droplet microarray printer. The printer was able to generate high‐quality picoliter droplets according to the preset programs and offer real‐time image feedback which allows a highly reproducible and large‐scale dispensing regime that conventional methods can hardly achieve (details were presented in “Methods”). First, we investigated 10 typical D‐amino acids (D‐Tyr, D‐Trp, D‐Met, D‐Leu, D‐Phe, D‐Pro, D‐cysteine (D‐Cys), D‐arginine (D‐Arg), D‐asparagine (D‐Asn), and D‐glutamic acid (D‐Glu)), which have been combined or used alone to combat biofilms in previous studies.^[^ ^33^, ^34^, ^35^, ^36^, ^37^ ^]^ The anti‐biofilm efficiency of these D‐amino acids at a concentration of 100 µM was detected by staining the biofilm of adhered cells with crystal violet. As shown in Figure S2 (Supporting Information), five D‐amino acids (D‐Tyr, D‐Trp, D‐Leu, D‐Phe, D‐Pro) out of ten were identified to show higher anti‐biofilm efficiency. Therefore, the aTyr‐bTrp‐cLeu‐dPhe‐ePro mixture was chosen to be optimized. The machine learning input dataset was formed from 96 single, binary, ternary, quaternary and pentabasic mixtures of D‐amino acids in a total concentration of 100 µM. The potential mixture space was defined as follows (the concentration is represented in D‐amino acid percentage): 0% ≤ a,b,c,d,e ≤ 100%, and each D‐amino acid in this space is constrained by a + b + c + d + e = 100% (100 µM). The details of the mixture arrangement were shown in Table S1 (Supporting Information). The OD_570_ value of the crystal violet solution was used to evaluate the anti‐biofilm performance of D‐amino acid mixtures. Finally, we obtained 96 data points (details were presented in “Experimental Section”) in the input dataset for further studies, with each point containing the ratio of a D‐amino acid mixture and its corresponding OD_570_ value.

### 2.3 Models Selection

**Figure 2:**
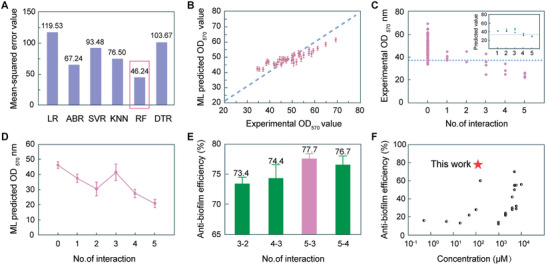
Results and evaluations of regression models and the global optimization. A) The MSE values for LR (logistic regression), ABR (AdaBoost regression), SVR (support vector regression), KNN (k‐nearest‐neighbors), RF (random forest regression), DTR (decision tree regression) models. B) The performance of the best regression model (RF) on the testing dataset for the anti‐biofilm property of D‐amino acid mixtures. C) The experimental measurements and machine learning predictions (inset) for OD_570_ values as a function of iteration number. At iteration 1–5, four new predicted D‐amino acid mixtures were measured. The blue dashed line represents the lowest value in the original input dataset. D) The MSE values of the model changed with the number of iterations. E) The anti‐biofilm properties of the best four D‐amino acids mixtures in our design loop. F) The comparison of the anti‐biofilm efficiency against *Pseudomonas aeruginosa* with the D‐amino acids reported in the literature.^[^ ^5^, ^18^, ^34^, ^43^, ^44^ ^]^

Based on the experimental data obtained by the methods described above, six regression models (logistic regression, AdaBoost regression, support vector regression, k‐nearest‐neighbors, random forest and decision tree) were built with the formulation of D‐amino acid mixtures as the descriptors to predict the anti‐biofilm performance. We employed the mean‐squared error (MSE) to evaluate the regression models and selected the one with the smallest MSE to train our input dataset. The MSE values for different regression models were shown in **Figure** **2**A. Among the six models, RF (random forest) yielded the best regression performance with an MSE of 46.24. The performance of the RF model on the input dataset of OD_570_ values was shown in Figure 2B and Figure S3 (Supporting Information). As shown in Figure 2B, the real values (abscissa of axis) were collected from the crystal violet staining experiment and the machine learning predicted (axis of ordinate) values were obtained by the RF model. A 45° blue dashed line represented that the real values and the predicted values were the same. Most of data points were situated close to the dashed line, indicating that the RF model had the ability to predict the anti‐biofilm performance of unexplored D‐amino acid mixtures. According to Figure S3 (Supporting Information), the deviation of the predicted values is concentrated within ± 6.3 of the real values, which is very small compared with the range of real values that exceeds 100.

### 2.4 Global Optimization

The regression studies are prone to reach local extrema because of the use of small datasets. They are simply guided by the output values of the model without exploring the search space where the uncertainties are largest.^[^ ^38^ ^]^ Thus, an adaptive approach that can guide the next experiments was needed to improve the quality of the regression model.^[^ ^39^, ^40^ ^]^ We adopted an EGO algorithm to select the next D‐amino acid mixture for experimental measurements by using the optimization index EI.^[^ ^41^ ^]^ This algorithm targets the regions of greater uncertainties rather than searching the potential D‐amino acid mixtures with higher anti‐biofilm efficiencies. EI was calculated by the following equation (1):

(1) $$\mathrm{EI}\;=\sigma \left[\phi \left(z\right)+z\Phi \left(z\right)\right]$$

in which z = (µ*‐µ)/σ, and µ* is the minimum value observed in the original input dataset. ϕ(z) and Φ(z) are the standard normal density and distribution functions, respectively. In detail, bootstrap sampling was used to evaluate the predicted value and the associated uncertainty of the D‐amino acid mixtures. We obtained 1000 predicted OD_570_ values for each D‐amino acid mixture in the unexplored composition space. The EI value for each D‐amino acid mixture was estimated by the mean values and standard deviation of the 1000 predictions. The D‐amino acid mixture with the largest EI value was one of the potential candidates for the experiment and selected to put into the original input dataset with its predicted value. Subsequently, a new input dataset including the original dataset and the new addition was trained to refine the model to yield another D‐amino acid mixture with the largest EI value which was chosen as the next candidate. We repeated this procedure (called “loop”) four times and finally obtained four candidates for experimental measurement (Details are given in Methods).

### 2.5 Experimental Iterative Feedback

Four new D‐amino acid mixture candidates were measured and the results of which were fed back to augment the input dataset (called “round”). We performed five design iterations and depicted how the iterative feedback loop worked (Figure 2C). The experimentally measured D‐amino acid mixtures from the iterative loop are given in **Table** **1**. The number 0 of iteration represents the OD_570_ values of the original input dataset. The gap between maximum and minimum OD_570_ values was large in the second iteration and became smaller in the subsequent iterations. In the third iteration, D‐amino acid mixtures with the OD_570_ values lower than 33.85 (the minimum OD_570_ value in the original input dataset) were identified. The result indicated that this loop could lead to the discovery of D‐amino acid mixtures with enhanced anti‐biofilm performance. The loop was executed iteratively until the OD_570_ values of the experimental D‐amino acid mixtures no longer decreased significantly, which eventually led to the discovery of the 15Tyr‐15Trp‐60Leu‐10Phe‐0Pro mixture (hereinafter referred to as D‐mix). As shown in Figure 2D, the MSE values of the model varied during experimental iterative feedback. On the whole, the MSE values of the regression model have decreased obviously after augmenting the input dataset guided by the iterative feedback loop. It was interesting to find that the quality of the regression model seemed to deteriorate after the third iteration. This trend could be interpreted as a consequence of the global optimization strategy, which prevents the model from being confined to local minima.^[^ ^27^ ^]^ Subsequently, we provided the curve of cumulative distribution function (CDF) versus prediction error to show the in vitro validation rate of D‐amino acid mixtures. The probability that the prediction deviation of the refined model is less than 4 is 92% (Figure S4, Supporting Information). These results indicated that the regression model has been refined during the iteration to have a higher capability of discovering a globally optimal D‐amino acid mixture.

**Table 1:** The real OD_570_ value and EI value of D‐amino acid mixtures predicted from the iterative design loop by machine learning.

No. of interaction^a^ ^)^	Cocktails	OD_570_	EI
**1‐1**	**0Tyr‐15Trp‐65Leu‐15Phe‐5Pro**	**40.9105**	**0.2718**
**1‐2**	**15Tyr‐55Trp‐25Leu‐0Phe‐5Pro**	**37.3587**	**0.1959**
**1‐3**	**0Tyr‐20Trp‐55Leu‐25Phe‐0Pro**	**44.7203**	**0.2473**
**1‐4**	**20Tyr‐65Trp‐15Leu‐0Phe‐0Pro**	**38.8357**	**0.2042**
**2‐1**	**0Tyr‐20Trp‐35Leu‐0Phe‐45Pro**	**64.8573**	**0.1369**
**2‐2**	**20Tyr‐60Trp‐20Leu‐0Phe‐0Pro**	**44.9213**	**0.0539**
**2‐3**	**0Tyr‐20Trp‐60Leu‐20Phe‐0Pro**	**35.8255**	**0.1229**
**3‐1**	**0Tyr‐15Trp‐60Leu‐25Phe‐0Pro**	**35.8954**	**0.0788**
**3‐2**	**10Tyr‐5Trp‐70Leu‐15Phe‐0Pro**	**26.5428**	**0.1226**
**3‐3**	**20Tyr‐0Trp‐60Leu‐20Phe‐0Pro**	**33.0864**	**0.1199**
**3‐4**	**0Tyr‐20Trp‐40Leu‐0Phe‐40Pro**	**42.8806**	**0.0783**
**4‐1**	**5Tyr‐20Trp‐55Leu‐20Phe‐5Pro**	**28.9823**	**0.0495**
**4‐2**	**25Tyr‐0Trp‐55Leu‐20Phe‐0Pro**	**34.2468**	**0.1411**
**4‐3**	**5Tyr‐25Trp‐50Leu‐20Phe‐0Pro**	**25.5547**	**0.0618**
**5‐1**	**5Tyr‐15Trp‐55Leu‐20Phe‐5Pro**	**27.1743**	**0.0539**
**5‐2**	**5Tyr‐25Trp‐50Leu‐20Phe‐0Pro**	**25.5907**	**0.0602**
**5‐3**	**15Tyr‐15Trp‐60Leu‐10Phe‐0Pro**	**22.3082**	**0.0190**
**5‐4**	**15Tyr‐15Trp‐55Leu‐10Phe‐5Pro**	**23.3065**	**0.0378**

^a)^ As for the No. of interactions of each D‐amino acids mixture, the first number indicates the iteration round, and the second represents D‐amino acids mixtures with the highest EI value in their respective design loop, e.g., No. of interaction 5–3 is the D‐amino acids mixture discovered in the third design loop of the fifth round.

We then ranked these five D‐amino acids in order of relative importance using the RF model.^[^ ^42^ ^]^ D‐Leu exhibited the highest importance for the anti‐biofilm performance of the mixture while D‐Pro was the least important (Figure S5, Supporting Information). The relative importance index of D‐Leu was 0.23 in the original regression model and significantly increased to 0.48 with the regression model refined by the EGO algorithm. The iterative feedback loop improved the regression model which became more capable of identifying the dominant role of D‐Leu in the mixture. We eventually found 7 D‐amino acid mixtures with the OD_570_ value lower than 33.85 out of the 18 experimental measured D‐amino acid mixtures from five rounds of feedback loops. The biofilm formation of all the experimental feedbacks was evaluated by crystal violet staining and four D‐amino acids mixtures with the best properties were listed (Figure 2E). The lowest OD_570_ value (∼22) obtained from D‐mix surpassed the best OD_570_ value in the original input dataset by ≈35%. D‐mix, discovered in the third design loop of the fifth round, possessed an anti‐biofilm efficiency of ≈78% with a concentration of 100 µM, which was the highest of the values reported in this study and also of the D‐amino acids or their mixtures reported in the literature (Figure 2F and Table S2, Supporting Information).

### 2.6 Identification of The Lowest Effective Concentrations of Antibiotics Based on High‐Throughput Experiments

Synergistic and potentiative drug combinations have attracted increasing attention because of their advantages such as enhanced antimicrobial efficacy, decreased dosage requirement, delayed development of AMR and reduced toxicity.^[^ ^45^, ^46^ ^]^ Developing an antimicrobial cocktail composed of a D‐mix and an antibiotic was expected to be a high‐efficacy and low‐toxicity treatment for bacterial infection. The microarray printer was employed for high‐throughput screening of drug combinations. 14 clinically verified antibiotics chosen from various categories were tested, including β‐lactam antibiotics (carbenicillin, ampicillin, amoxicillin, meropenem, chloramphenicol), cephalosporins antibiotics (cefotaxime, ceftazidime), aminoglycoside antibiotics (gentamicin, kanamycin, streptomycin), tetracyclines antibiotics (tetracycline), antimicrobial peptides (polymyxin B), sulfonamides antibiotics (sulfamethoxazole), and macrolide antibiotics (erythromycin). As a human facultative pathogenic bacterium, *P. aeruginosa* was recognized for its ubiquity and intrinsically advanced antibiotic resistance mechanisms.^[^ ^47^, ^48^ ^]^ Therefore, the minimum inhibitory concentrations (MICs) of *P. aeruginosa* obtained from the previous research (Table S3, Supporting Information) may not be applicable for the current study.^[^ ^49^ ^]^ Moreover, Kaplan et al. discovered the sub‐inhibitory concentrations of antibiotics would induce extracellular DNA release and promote biofilm formation.^[^ ^50^ ^]^ As such, rapidly assessing the effective inhibitory concentrations of each antibiotic against *P. aeruginosa* was critical to the subsequent identification of D‐mix and antibiotics synergies. We simultaneously measured the growth inhibition rate of the antibiotics to *P. aeruginosa* at concentrations ranging from 1‐fold to 100‐fold MIC. Antibiotics without obvious inhibition effect on the bacteria at the concentrations of 100‐fold MIC were considered to be not sensitive (NS). The concentrations capable of inhibiting bacterial growth by 90% over a 1‐day period as determined via the OD_600_ value were considered as the LEC for each antibiotic. The inhibition rates of 14 different kinds of antibiotics against *P. aeruginosa* at various concentrations were shown in Figure S6, Supporting Information, while their LEC was summarized in Table S4 (Supporting Information). Chloramphenicol and sulfamethoxazole were defined as not sensitive (NS) because no obvious inhibitory effect against bacterial growth was observed in the test range. We left out chloramphenicol and sulfamethoxazole in this work and screened 12 antibiotics for the subsequent tests.

### 2.7 In Vitro Antimicrobial Activity Screening of D‐Amino Acids and Antibiotics Cocktails

**Figure 3:**
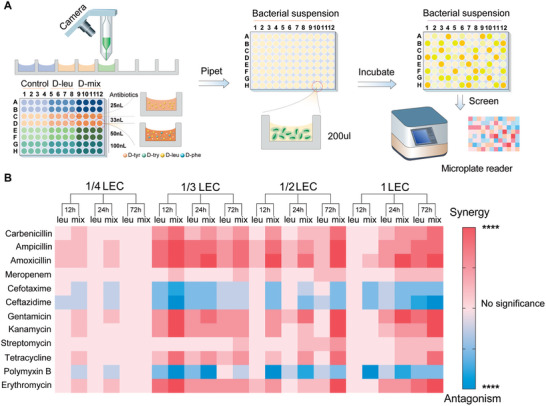
In vitro antimicrobial activity screening. A) Schematic diagram showing the high‐throughput screening process. B) Heat map of the antimicrobial synergy/antagonism of D‐amino acids and antibiotics cocktails based on high‐throughput screening assays. Two‐way analysis of variance was used for statistical analysis. Data are means ±SD (n = 4, SD indicates standard deviation, * indicates p < 0.05, ** indicates p < 0.01, *** indicates p < 0.001, and **** indicates p < 0.0001).

Using the droplet microarray printer, we evaluated the antimicrobial behaviors of drug combinations under 288 conditions, involving 12 antibiotics, 4 concentrations (1/4 LEC, 1/3 LEC, 1/2 LEC and 1 LEC), three time intervals (12, 24, and 72 hours), and two kinds of anti‐biofilm agents (D‐mix and D‐leu) (**Figure** **3**). The data visualization was achieved by summarizing the degree of statistical differences in the antimicrobial efficiencies between drug combinations and antibiotics alone. Figure 3B shows a heat map that rates the synergy (or antagonism) between the D‐amino acids and antibiotics on the inhibition of bacterial growth in a 12 × 24 matrix, where blue represents antagonism and red represents synergy. The depth of color is determined by the level of statistical differences using the two‐way analysis of variance . It is worth noting that, with the introduction of D‐amino acids, not all antibiotics were enhanced in their antimicrobial efficiency. In the presence of D‐mix, 9 antibiotics exhibited enhanced antibacterial performance. With lower dosages of the antibiotic (1/3 LEC or 1/2 LEC), the antimicrobial performance of ampicillin/D‐mix and amoxicillin/D‐mix had the most significant improvement compared to the antibiotics alone (evidenced by the largest number of deepest red squares visible). Gentamicin/D‐mix cocktails stood out in a higher range of antibiotic dosages (1 LEC). According to Rahman et al., D‐amino acids target on the matrix component extracellular DNA and interact with positively charged bacterial surfaces, thereby preventing the formation of intercellular bridges and weakening the resistance of bacteria to antibiotics.^[^ ^21^ ^]^ Horswill et al. claimed D‐amino acids could aid the exposure of metabolically active bacteria and render remaining persister bacteria vulnerable for the actions of antibiotics.^[^ ^51^ ^]^ Besides the synergistic D‐amino acids and antibiotics mixtures, we also screened out 3 antibiotics that were not suitable to cooperate with D‐mix, among which polymyxin B exhibited strong antagonism to D‐mix. This finding agrees with the work by Nantasenamat et al., which showed that the incorporation of D‐amino acids inactivated the antimicrobial activity of cyclic polymyxin B.^[^ ^52^ ^]^ It is worth noting that, the combined behaviors (Figure 3B) of antibiotics and D‐mix are not directly related to the category of antibiotics (Table S4, Supporting Information). For example, ampicillin and amoxicillin exhibited a synergistic effect with D‐mix while cefotaxime and ceftazidime went antagonistic although these four are all β‐lactamase antibiotics. As is well known, β‐lactamase antibiotics refer to an organic structure containing a lactam ring. However, apart from the lactam ring, the structures of cephalosporins and ampicillin or amoxicillin have significant differences. As shown in Table S5 (Supporting Information), we implemented some physicochemical descriptors (Labute) molecular, graph descriptors (Chi0, HallKierAlpha), hydrogen‐bonding descriptors (HBA), and 3D shape descriptors (FracCSP3, PMI3, SpherocityIndex, RadiusOfGyration) of ampicillin, amoxicillin, cefotaxime and ceftazidime in the *RDKit* library. All descriptor values are relatively close between ampicillin and amoxicillin or between cefotaxime and ceftazidime, but have a large discrepancy between ampicillin and cephalosporins. These hidden characteristics of antibiotics may play a crucial role was important in their specificity towards D‐mix. Moreover, we observed that the D‐mix always exhibited a greater impact (either synergy or antagonism) on the antibiotics than D‐leu alone in this work. Overall, we successfully screened out the antibiotics that exhibited significantly improved antimicrobial performance in cooperation with D‐mix and verified the synergy of drug combinations.

**Figure 4:**
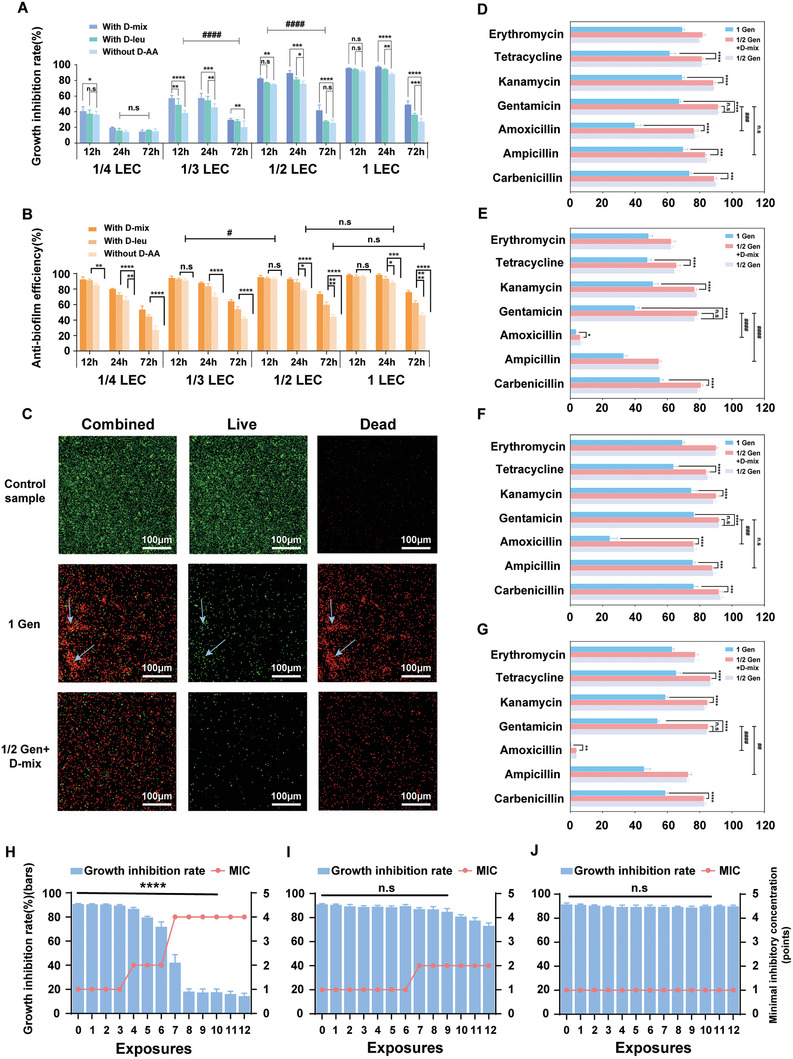
High‐throughput antimicrobial activity and toxicity evaluation. A) The growth inhibition rates and B) anti‐biofilm efficiency of *P. aeruginosa* via combination therapy with different gentamicin dosages at 12, 24, and 72 hours. C) Fluorescence microscopic images of *P. aeruginosa*. The blue arrows point to the bacteria encapsulated by EPS. The toxicity to BESA‐2B cell lines measured by CCK‐8 assay after D) 24 hours and E) 72 hours of incubation. The toxicity to AML‐12 cell lines measured by CCK‐8 assay after F) 24 hours and G) 72 hours of incubation. The antimicrobial behavior of H) gentamicin alone, I) gentamicin/D‐mix cocktail and J)D‐mix alone after different number of exposures (exposure 0 represents the *P. aeruginosa* prior to treatment with any of the drugs or drug combinations in this work). Two‐way analysis of variance was used for statistical analysis. Data are means ±SD (n = 4, SD indicates standard deviation; * indicates p < 0.05, ** indicates p < 0.01, *** indicates p < 0.001, and **** indicates p < 0.0001). The statistical differences relating to D‐mix and antibiotic combinations with different incubation times are represented by doubles symbols (# indicates p < 0.05, ## indicates p < 0.01, ### indicates p < 0.001, and #### indicates p < 0.0001).

According to the screening results, we evaluated the antimicrobial performance and anti‐biofilm efficiency of gentamicin/D‐amino acid cocktails. As shown in **Figure** **4**A, 1/2 LEC dosages of gentamicin coupled with D‐mix were able to guarantee an excellent antimicrobial performance (≈91%) during the period of 24 hours, which was equivalent to using 1 LEC gentamicin alone (≈90%). However, no bactericidal activity was detected when 100 µM D‐mix was used alone (Figure S7, Supporting Information). Therefore, the enhanced antimicrobial activity of combinations was attributed to the significant anti‐biofilm properties of D‐mix, which endowed the combination with ≈93% anti‐biofilm efficiency (Figure 4B). Without the obstinate defense of the biofilm, 1/2 LEC gentamicin was enough to effectively inhibit bacterial growth. In order to further study the role of D‐mix, confocal laser scanning microscopy (CLSM) was used to detect the bacterial distribution on the surface. As shown in Figure 4C, when exposed to 1/2 LEC gentamicin/D‐mix cocktails, the cells were highly scattered due to the lack of an organized biofilm structure. In comparison, cells treated with gentamicin alone were encapsulated by interconnected biofilm matrices (marked with blue arrows) and distributed in large clusters. Similarly, cooperating with D‐mix, the antimicrobial efficiency of 1/2 LEC ampicillin and 1/2 LEC amoxicillin was also significantly enhanced to 86% and 91% (Figure S8, Supporting Information), respectively. Overall, synergistic gentamicin/D‐mix and amoxicillin/D‐mix cocktails were identified to maintain an equal or increased level of efficacy (≥90%) with decreased antibiotic dosage requirements.

### 2.8 Toxicity Evaluation of Antibiotic/D‐Mix Cocktails

Owing to defective drug metabolism or excessive dosing regimen, clinical antibiotics may damage human organs via crystallization, immune response and high concentrations induced toxicity.^[^ ^53^, ^54^ ^]^ Moreover, clinical antibiotics may trigger oxidative stress (formation of reactive oxygen molecules) in mitochondria, resulting in cell toxicity to mammals. Owing to the similarity between human and mammalian mitochondria, some antibiotics may severely damage human organ cells via inhibiting the function of mitochondria.^[^ ^55^, ^56^ ^]^ The strategy of drug combinations could effectively reduce toxicity by lowering the dosage requirement for antibiotics.^[^ ^45^ ^]^ We selected 7 antibiotics that had a relatively strong synergistic effect with D‐mix and evaluated the toxicity of them and their combination with D‐mix to human lung epithelial (BEAS‐2B), mouse liver (AML‐12) and mouse pre‐osteoblastic (MC3T3‐E1) cell lines. Figure 4D–G and Figure S9 (Supporting Information) demonstrated the toxicity screening results of the Cell Counting Kit‐8 (CCK‐8) assay after 24 and 72 hours of incubation. Take the BEAS‐2B cell line as an example (Figure 4D,E). When used alone, these 7 antibiotics extremely harmed the cell lines, resulting in a substantial reduction in the BEAS‐2B cell viability to 3.4%−55.2% in 3 days. The dosage of 1 LEC amoxicillin killed almost all cells. We also investigated the toxicity of 1/2 LEC antibiotics cooperated with 100 µM D‐mix. Compared to using antibiotics alone, the cell viability values were significantly increased for all the drug cocktails and remained ≈80% for two of them even after 72 hours. The gentamicin/D‐mix cocktails exhibited the highest cell viability among all samples, 92% after 24 hours and 80% after 72 hours. In contrast, gentamicin itself exhibited considerable toxicity (only 40% cell viability in 3 days) to BEAS‐2B cells. These results indicated that once we reduced the concentration of gentamicin to 4 mg/L (1/2 LEC) and used 100 µM D‐mix as a supplement, the toxicity of gentamicin and D‐mix cocktails would be reduced to a very low level. As shown in The same phenomenon was indicated in the toxicity evaluation to mammalian cells (AML‐12 and MC3T3‐E1), gentamicin/D‐mix cocktails exhibited high cell viability to AML‐12 (≈91%) and MC3T3‐E1(≈96%) cell lines after 24 hours. Therefore, gentamicin coupled with D‐mix was identified as the optimal combination in this study with superior antimicrobial performance yet almost no toxicity to combat microbial infection. We further investigated the ability of the bacteria to develop resistance to gentamicin/D‐mix cocktails, D‐mix or gentamicin alone (Figure 4H–J). After the 4^th^ exposure with gentamicin alone, the bacteria began to show significant resistance. Only after the 8^th^ exposure, 1 LEC gentamicin almost lost its antibacterial properties. The MIC of gentamicin increased to 4 LEC (32 mg L^−1^). In comparison, the development of resistance in bacteria treated with the gentamicin/D‐mix cocktail was significantly delayed. The bacterial growth inhibition rate of 1/2 LEC gentamicin/D‐mix cocktails remained at ≈85% after the 8^th^ exposure, and the MIC only increased to 2 LEC even after the 12^th^ exposure treatment. These results clearly demonstrated that lowering the dosage of gentamicin (1/2 LEC) and using D‐mix as a biocompatible enhancer (no significant development of drug resistance was observed in D‐mix treated bacteria) could effectively combat AMR.

### 2.9 In Vivo Assays and Hemolytic Activities of The Optimal Antimicrobial Cocktails

**Figure 5:**
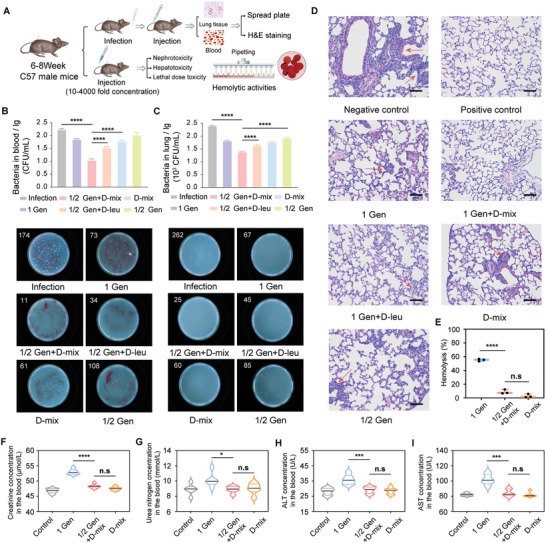
A) Schematic diagram showing the process of in vivo assays and hemolytic activities. Colony forming units (CFUs) of *P. aeruginosa* in B) blood and C) lung at 72 hours post infection (n = 3), the white number in the upper left corner represents the quantity of visible colonies on the plates. D) Hematoxylin‐eosin (H&E) stained images for lung tissue with different drug treatments at 72 hours post infection. E) Percentage of hemolysis of rRBCs (n = 3) F) Creatinine and G) Urea nitrogen concentration in the blood (n = 6) after injecting different drugs at 24 hours. H) ALT and I) AST nitrogen concentration in the blood (n = 6) after injecting different drugs at 24 hours. Two‐way analysis of variance was used for statistical analysis. Data are means ±SD (SD indicates standard deviation; * indicates p < 0.05, ** indicates p < 0.01, *** indicates p < 0.001, and **** indicates p < 0.0001).

The in vivo treatment efficacy of the D‐amino acid/antibiotic cocktails was then demonstrated in a *P. aeruginosa*‐induced lung infection mouse model by intranasal instillation (**Figure** **5**A). Bacteria were counted in the blood (Figure 5B) and lung tissue (Figure 5C) after various drug treatments (LEC of the optimal antimicrobial cocktails against *P. aeruginosa*: Gentamicin coupled of 4 mg L^−1^with 100 µM D‐mix; 4 mg k^−1^g gentamicin coupled with 100 µmol kg^−1^ D‐mix of mouse body weight per injection, 3 i.v. injections at 1, 8, and 24 hours post infection). Among these treatments, the gentamicin /D‐mix cocktails were most effective in removing the bacteria from the blood (≈95%) and the lung tissues (≈91%) at 72 hours post infection. In comparison, treating with gentamicin alone or gentamicin/D‐leu cocktails all exhibited significantly lower antimicrobial efficacy under in vivo evaluation. In addition, we used the hematoxylin‐eosin (H&E) staining of lung tissue treated with different therapies at 72 hours post infection. As shown in Figure 5D, H&E stained sections of the infected lung tissue treated with D‐mix/gentamicin cocktails achieved no pathological changes, which was comparable to positive control groups (without bacterial infection). In contrast, mice in the other lung infection model groups exhibited varying degrees of thickened alveolar wall and inflammatory cell infiltration in the alveolar septum (marked with red arrows). These results demonstrated the superior in vivo antimicrobial efficacy of gentamicin/D‐mix cocktails and their treatment has reversed the damage induced by *P. aeruginosa*.

We further performed the in vivo toxicity test (Figure 5A). The Spearman‐Karber method^[^ ^57^ ^]^ was employed to estimate the lethal dose (LD_50_) toxicity values based on the survival curve of the drug injected mice over 1 day (Figure S10, Supporting Information). The LD_50_ values of D‐mix–gentamicin cocktails (750 mg k^−1^g) were much higher than using gentamicin alone (345 mg k^−1^g). Notably, D‐mix itself did not cause any mouse death even at the highest tested concentration (2,430 mg k^−1^g). The introduction of D‐mix tremendously reduced the extent of hemolysis of the cocktails (Figure 5E). Moreover, the gentamicin/D‐mix cocktails did not induce any nephro‐ (Figure 5F,G) or hepato‐toxicity (Figure 5H,I). The in vivo study also confirmed that lowering the dosage requirement for gentamicin and using D‐mix as a supplement significantly decreased the toxicity of the antimicrobial therapy.

## 3 Discussion

The discovery of high‐performing antibiotic/D‐mix cocktails based therapies to treat AMR from bacterial pathogens is difficult and occasional due to the gigantic search space of the compositions.^[^ ^58^, ^59^, ^60^ ^]^ High‐throughput techniques have been widely used for combinatorial screening of small‐molecule drugs and polymer materials.^[^ ^61^, ^62^ ^]^ However, they are only capable of discovering drugs within the limited experimental results and cannot obtain any information beyond the experimental parameters space. In this work, the possible number of D‐amino acids and antibiotics combinations is too large for optimization solely through high‐throughput experiments. Machine learning algorithms allow the prediction of new drug formulations outside the original libraries, making up for the limitations of high‐throughput techniques. Herein, we demonstrated an efficient methodology that combines high‐throughput experiments and machine learning optimization for the design of potent antimicrobial drug combinations and validated the efficacy and toxicity of these drugs via both in vitro and in vivo studies. First, an automated droplet microarray printer was used to construct an input dataset of D‐amino acid and their mixtures for machine learning models. The automated microarray printer takes less than one second to perform a single dispensing at a picoliter level, which allows the construction of the entire mixture library in a 96‐well plate within only 10 minutes. Second, we employed a global optimization algorithm (EGO) to greatly narrow the searching space of the D‐amino acids based on the EI values. Benefiting from the EGO, the number of possible D‐amino acid combinations requiring experimental investigation on their anti‐biofilm performance was reduced from tens of thousands to only 18. During machine learning training, we took the concentration ratios as the descriptor of different D‐amino acids in mixtures. With the guide of EGO, we explored the mixture space where the uncertainties are largest. It is an adaptive procedure that makes optimal choices of D‐amino acid mixtures to test next by balancing the merits of searching for mixtures likely to have the best anti‐biofilm property or where there may be fewer sampling points. The advantage of this experimental selection method is that it provides robust and rapid navigation in unexplored space by maximizing the expected improvement from the best‐so‐far mixtures. In fact, regression studies are prone to predict suboptimal D‐amino acid mixtures hampered by small data sets or large models. The EGO and experimental feedback aid us to avoid some suboptimal possibilities (such as 15Tyr‐55Trp‐25Leu‐0Phe‐5Pro or 0Tyr‐20Trp‐40Leu‐0Phe‐40Pro), and finally guide us to the global optimal recipe. This machine learning design loop also improves the quality of the regression model in the long run (Figure 2; Figure S5, Supporting Information). Subsequently, we employed the same high‐throughput screening platform and successfully identified the in vitro efficacy of the mixture between D‐amino acids and various antibiotics (Figure 3B). The discovered gentamicin/D‐mix cocktail demonstrated high antimicrobial activity at low concentrations, as well as the ability to delay AMR onset (Figure 4F).

Cells exposed to D‐amino acid mixtures were more significantly impaired in biofilm formation than D‐amino acids alone.^[^ ^16^, ^17^ ^]^ Thus, D‐amino acids may act synergistically. Such synergism may be attributed to the different targets of each D‐amino acid on peptidoglycan. Specifically, bacteria have been found to produce diverse D‐amino acids during incubation. For example, *V. cholerae* could produce D‐Met, D‐Leu, D‐Val, and D‐Ile in stationary phase supernatants.^[^ ^63^ ^]^ These D‐amino acids would modulate the synthesis and strongly influence the composition, amount and strength of peptidoglycan to adapt to the environment. The exogenous D‐amino acids probably disturbed this synthesis by incorporation into the stationary phase of peptidoglycan, resulting in a dramatic alteration in the strength or flexibility of peptidoglycan. The anti‐biofilm performance of exogenous D‐amino acids is attributed to these alterations.^[^ ^64^ ^]^ Notably, multiple exogenous D‐amino acids are involved in the regulation of peptidoglycan structure simultaneously.^[^ ^58^ ^]^ D‐mix could alter multiple positions of the peptidoglycan‐peptide bridge by incorporation into the polymer to maximize the inhibition of *P. aeruginosa* biofilm formation. However, the mechanisms behind the synergism also remain largely unclear. In this case, machine learning algorithms may be helpful in optimizing D‐amino acid mixtures by “learning” from the data on their own to provide active guidance in the discovery of potent formulations.

We systematically investigated the in vitro antimicrobial activity of every D‐mix/antibiotics cocktail. The results revealed that D‐mix served as an enhancer for most antibiotics, which results from the decent anti‐biofilm performance of D‐mix. We replaced the D‐amino acids of the optimal mixture with the corresponding L‐amino acids, finding that the L‐mix in combination therapy did not obviously enhance the antimicrobial performance of gentamicin (Figure S11, Supporting Information). These results proved that D‐stereochemistry of the amino acids was important for the cocktail therapy. However, D‐mix could also reduce the efficacy of some antibiotics (cefotaxime, ceftazidime and polymyxin B) when their integration into the peptidoglycan changes the primary target for the antibiotic.^[^ ^65^, ^66^ ^]^ Furthermore, optimal cocktails were demonstrated to maintain an equal or increased treatment efficacy in vitro with significantly lowered antibiotic dosage requirements (Figure 4A). Notably, the same findings were identified in the lung infection mouse study, which highlighted the potential of D‐mix as an in vivo therapeutic candidate to enhance the performance of antibiotics. The innovative design strategy in this study could rapidly explore the vast drug combination space and achieve large‐scale and reliable screening of not only AMR therapeutics but also other biomedicines and biomaterials.

## 4 Experimental Section

### Materials

All D‐amino acids (D‐Tyr, D‐Trp, D‐Met, D‐Leu, D‐Phe, D‐Pro, D‐Cys, D‐Arg, D‐Asn and D‐Glu) and all antibiotics (gentamicin sulfate, carbenicillin, ampicillin, tetracycline hydrochloride, cefotaxime acid, ceftazidime, chloramphenicol, erythromycin, amoxicillin, kanamycin sulfate, streptomycin sulfate, polymyxin B sulfate, meropenem, sulfamethoxazole) were purchased from Aladdin Industrial Corporation. Crystal violet, cyclophosphamide, hematoxylin and eosin were purchased from Sigma‐Aldrich. All chemicals were used as received without further purification. The *P. aeruginosa* (ATCC 15 692) strains were obtained from China General Microbiological Culture Collection Center, Beijing, China. The cell lines, the complete medium and the minimum essential medium used in this study were obtained from Procell Life Science & Technology *Co., Ltd*, Wuhan, China. The high glucose DMEM medium was obtained from Corning Incorporated, USA.

### High‐Throughput Droplet Dispensing

The dispensing was carried out based on a versatile non‐contact droplet microarray printer (Nano‐PlotterTM NP2.1, Germany), which was equipped with piezoelectric pipetting tips for the software‐controlled deposition of particular drop bursts. The samples were aspirated from 96/384 well microplates and an automatic wash/dry station was triggered subsequently. The dispensing parameters were optimized and verified before the procedure started. The solenoid valve dispensers of microarray printers dispense exact volumes of liquids into various plates or surfaces and offer real‐time imaging of the mixed drops. Drops with the volumes as tiny as picoliters can be quickly and precisely dispensed to the pre‐arranged location. The humidity was maintained at 80% during dispensing.

### Bacterial Growth

Cells were cultured in a shaking Luria‐Bertani (LB) fluid medium at 37 °C overnight and diluted to 5×10^5^ CFU mL^−1^ for later use. The microarray printer was used to dispense pre‐determined amounts of five kinds of D‐amino in 96‐well polystyrene plates. Each well of the plate was pre‐loaded with 100 µL diluted LB solution. Generally speaking, the D‐amino acid solutions were prepared with high concentrations and dispensed a total volume of 100 nL of it to each well. The entire dispensing process for each 96‐well plate took only 10 min and required less than 10 µL solution in total. Then, the bacterial cells were grown for 18 hours in the bottom of 96‐well polystyrene plates without shaking at 37 °C for data collection. Multiple antibiotics and D‐amino acids were dispensed in the 96‐well plates via the microarray printer and incubated with LB medium containing 5×10^5^ CFU mL^‐1^ cells for 8, 24 and 72 hours for high‐throughput screening. The planktonic cells were removed by gently rinsing with phosphate‐buffered saline (PBS) before further treatments.

### Crystal Violet Staining

After rinsing in PBS twice, the adhered cells were stained with crystal violet. The wells were stained with 100 µL of 0.1% crystal violet dye, rinsed 3 times with 200 µL Milli‐Q water, and thoroughly dried overnight. For the quantification of biofilm growth, 200 µL 4% (w/v) sodium dodecyl sulfate (SDS) was added to the stained wells and shaken for 10 min for complete dissolution. The solution was diluted by a factor of 4 and its optical density was measured at 570 nm. Anti‐biofilm efficiency was calculated by the following equation (2):

(2) $$\mathrm{Anti}-\mathrm{biofilm}\;\mathrm{efficiency}=\left(C_{\mathrm{OD}}-E_{\mathrm{OD}}\right)/C_{\mathrm{OD}}\times 100\%$$

in which C_OD_ and E_OD_ were the OD_570_ values of the control group and experimental group, respectively.

### Input Dataset Construction

In order to ensure the reproducibility of the experimental results, The OD_570_ value of each D‐amino acid mixture was measured based on 10 replicates. Initially collected 970 data points were collected (97 well and 10 spots), including 960 experimental groups and 10 blank controls, and all results were normalized to a blank control (fixed at 100 OD_570_ in 96‐well plates). The average of these 10 replicates was used to construct the input dataset, which consisted of 96 data points.

### Machine‐Learning Models

Logistic regression, AdaBoost regression, support vector regression, k‐nearest‐neighbors, random forest and decision tree classification models were employed to train the original input dataset and the hyperparameters of each model were tuned by the bootstrap approach via cross‐validation. 80% of the original data were randomly chosen as a training set to construct the model and 20% of them were used as a testing set to evaluate the quality of the model. Each model was trained 100 times in pre‐arranged hyperparameters and the $\bar{\mathrm{MSE}}$ (the mean MSE value of these 100 models) was used to evaluate the quality of different regression models. MSE was calculated by the following equation (3):

(3) $$\mathrm{MSE}=\sum_{k=1}^{n}\left(y_{k}-{\dot{y}}_{k}\right)$$

in which y_k_ and ${\dot{y}}_{k}$were the experimental value and machine learning predicted value, respectively. The models with the minimal MSE value were selected for further study.

### Global Optimization Strategy

An adaptive procedure were adopted based on the powerful optimization strategy EGO to make optimal choices of D‐amino acid mixtures by maximizing the EI over the search space. As shown in equation (1), σ and µ* was the “best‐so‐far” OD_570_ value of D‐amino acid mixtures; it was assumed to be a minimum. The EI gives the improvements on the current lowest OD_570_ value by sampling D‐amino acid mixtures in the search space. The global optimization strategy would maximize the “expected improvement”, thus balancing the trade‐off between exploitation and exploration, and move onto regions of greater uncertainty at the expense of lower values of OD_570_, which accelerated the discovery of targeted mixtures. In this work, each component in D‐amino acid mixtures ranged from 0%−100%, and the step size was set to 5%.

### In Vitro Antimicrobial Efficiency Evaluation

Before the antimicrobial efficiency evaluation, the cells were incubated in a shaking LB fluid medium at 37 °C overnight. Various antibiotic and D‐amino acid solutions in this work were prepared with high concentrations and dispensed into 96‐well plates via the microarray printer. Each well in 96‐well plates was pre‐loaded 100 µL LB with 10^6^ CFU mL^−1^ cells suspension. The plates were incubated in 37 °C for 4, 8, 12, 24, 48, and 72 hours. A microplate reader (Multiskan FC, ThermoFisher Scientific, USA) was used to spectrophotometrically measure the absorbance of samples at 600 nm. The antimicrobial efficiency was calculated by the following equation (4):

(4) $$\mathrm{Antimicrobial}\;\mathrm{efficiency}=\left(1-E_{\mathrm{OD}}/C_{\mathrm{OD}}\right)\times 100\%$$

in which E_OD_ and C_OD_ were the OD_600_ values of the experimental group and the control group, respectively.

### Fluorescence Microscope Method

After exposure to *P. aeruginosa* for 24 hours, the stainless steel surface treated with different antimicrobial drugs was taken out of the bacterial suspension and washed with PBS several times to remove any planktonic cells. The cell‐adhered surfaces were stained with 20 µL STYO‐9 and propidium iodide (PI) dye (Invitrogen, Eugene, USA) for 30 min in the dark. Confocal laser scanning microscopy (CLSM) (Model C2 Plus, Nikon, Japan) was employed to observe the distribution of live/dead strains on the surfaces. SYTO‐9 and PI dye were excited as green fluorescence and red fluorescence at a wavelength of 488 and 559 nm, respectively.

### In Vitro Toxicity Evaluation

The BESA‐2B, AML‐12 and MC3T3‐E1 cell lines were used for in vitro toxicity evaluation via the CCK‐8 (Beyotime, China) assay. The BESA‐2B, AML‐12 and MC3T3‐E1 cells were cultured in a high glucose DMEM medium, a complete medium and   a minimum essential medium, respectively. The logarithmic phase adherent cells were detached by adding trypsin and inoculated into the 96‐well plate at a density of 5×10^4^ CFU mL^−1^. Antibiotics and their combinations at selected concentrations were then dispensed in 96‐well plates. After 24 and 72 hours, the culture solution was removed and a 100 µL medium with 10% CCK‐8 solution was added to each well. Solutions were cultivated at 37 °C for 4 hours in the dark. The OD values of each well were measured using a microplate reader at 450 nm. The cell viability of each combination was calculated using the following equation (5):

(5) $$\mathrm{Cell}\;\mathrm{viability}=\left(1-T_{\mathrm{OD}}/U_{\mathrm{OD}}\right)\;\times \;100\%$$

in which T_OD_ and U_OD_ were the OD_450_ values of the treated samples and the untreated samples, respectively.

### The Resistance Development Evaluation

Bacteria was continuously treated with gentamicin alone, gentamicin/D‐mix cocktail and D‐mix alone to capture the development of their resistance. In detail, the bacteria at exposure 0 were incubated in a shaking LB fluid medium at 37 °C overnight. Different concentrations of gentamicin, gentamicin/D‐mix cocktail and D‐mix alone were transferred into 96‐well plates via the microarray printer. Each well of plates was pre‐loaded 100 µL 10^6^ CFU mL^−1^ cells suspension. The plates were incubated at 37 °C for 24 hours. OD_600_ value and checkerboard method were used to evaluate the bacterial growth inhibition rate and MIC value of drugs, respectively. The bacteria was pipetted out that survived exposure to 1 LEC Gentamicin, 1/2 LEC gentamicin/D‐mix cocktail or 1 LEC (100 µM) and incubated them in fresh LB at 37 °C overnight. These bacteria were called ‘exposure 1′. This process was repeated until it finished the evaluation of the antimicrobial behavior of the drugs or cocktail against bacteria after the 8^th^ exposure.

### Lung Infection Model

C57BL/6J mice (female, 6–8 weeks, 20–30 g, Geneline Bioscience) were randomly divided into 6 groups (infection without any treatment, 1 Gen treated, 1/2Gen + D‐mix treated, 1/2Gen + D‐leu treated, D‐mix treated and 1/2 Gen treated, 6 mice in each group). The lung infection model was established by intranasal instillation of *P.aeruginosa* (ATCC 15 692) strain; [1× 10^7^ CFU mL^‐1^; 20 µL for each mouse]. 150 mg k^−1^g cyclophosphamide was injected subcutaneously 4 days before infection. The infected mice were treated with various drugs or drug combinations in this work at 1, 8, and 24 hours post infection (8 mg k^−1^g gentamicin of mouse body weight per injection for 1 Gen group, 4 mg k^−1^g gentamicin coupled with 100 µmol kg^−1^ D‐mix of mouse body weight per injection for 1/2Gen + D‐mix group, 4 mg k^−1^g gentamicin coupled with 100 µmol kg^−1^ D‐leu of mouse body weight per injection for 1/2Gen + D‐leu group, 100 µmol kg^−1^ D‐mix of mouse body weight per injection for D‐mix group and 4 mg k^−1^g gentamicin of mouse body weight per injection for 1/2 Gen group.

### In Vivo Antimicrobial Efficiency Evaluation

Three mice in each group were euthanized at 72 hours post infection. The blood and the lung tissue were collected for analysis of bacterial removing efficiency in vivo. The blood was mixed with EDTA solution in the presence of the anti‐coagulant heparin and stored at 4 °C before use. Then, the lung was taken out and homogenized in 2 mL of PBS. The blood and homogenate were diluted and plated on MH agar plates. After 18 hours incubation at 37 °C, the number of bacterial colonies was counted (Scan 1200, Interscience, France). The data were presented as CFU mL^‐1^ for blood and 10^3^ CFU mL^‐1^ for homogenate.

### Hematoxylin‐Eosin Staining

Another 3 mice in each group were euthanized at 72 hours post infection and their lung segments were collected, embedded in paraffin and sectioned for H&E staining assay. The lung tissue slices were dried at 40 ˚C and hydrated with a gradient of ethanol solutions. Subsequently, the slices were stained with hematoxylin and eosin. 200× images for each slice were captured using a microscope (Eclopse 80i, Nikon, Japan).

### Ethics Approval

All animal experiments were performed in accordance with the National Laboratory Animal Welfare Ethics and approved by the Animal Ethical and Welfare Committee (AEWC) of Geneline Bioscience (Beijing, China) (JL‐YJZ‐20220609).

## Conflict of Interest

The authors declare no conflict of interest.

## Author Contributions

D.Z. and J.Y. conceived the study. D.Z., P.J., and X.L supervised the research. J.Y. conducted most of the experiments, including machine learning design loop, high throughput screening process, and in vivo experiments. Y.R and C.R assisted in high throughput experiments and helped with the analysis of the results. S.L assisted in the machine learning model evaluation. Y.L helped with the in vivo infection models. J.Y. wrote the manuscript and designed the figures. All the authors participated in manuscript reviewing and editing.

## Supporting information

Supporting Information

## Data Availability

The data that support the findings of this study are available from the corresponding author upon reasonable request.
