# Supplementary material for: Synergistic D‐Amino Acids Based Antimicrobial Cocktails Formulated via High‐Throughput Screening and Machine Learning
Source: Adv Sci (Weinh). 2023 Dec 21;11(9):2307173. doi: 10.1002/advs.202307173 (PMC10916672; doi:10.1002/advs.202307173)
Supplement: Supplementary file 1 — Supporting Information [file ADVS-11-2307173-s001.pdf]

ADVANCED SCIENCE
Open Access

Supporting Information

for *Adv. Sci.*, DOI 10.1002/advs.202307173

Synergistic D-Amino Acids Based Antimicrobial Cocktails Formulated via High-Throughput Screening and Machine Learning

*Jingzhi Yang, Yami Ran, Shaopeng Liu, Chenhao Ren, Yuntian Lou, Pengfei Ju, Guoliang Li, Xiaogang Li and Dawei Zhang**

Supplementary information

Synergistic D-amino acids based antimicrobial cocktails formulated via high-throughput screening and machine learning

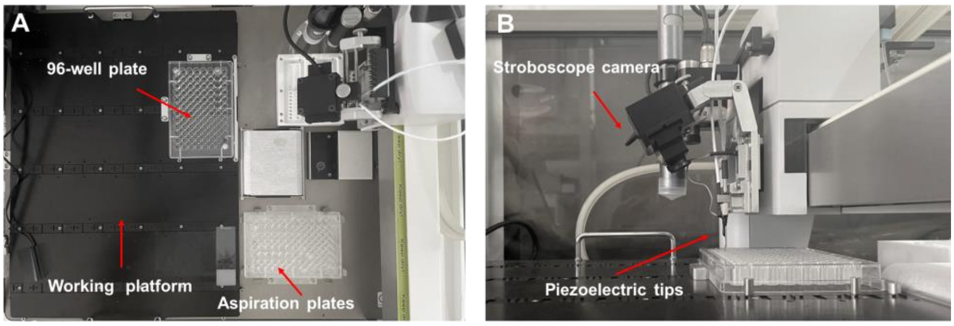

Supplementary Figure 1. The high-throughput experimental platform: (a) the top view; (b) the side view.

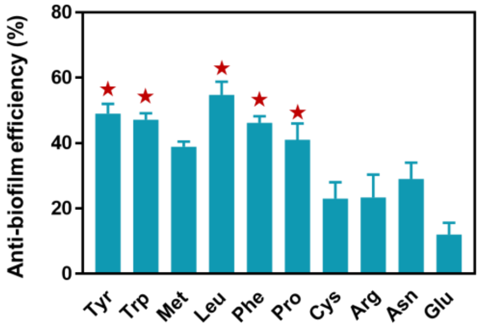

Supplementary Figure 2. The anti-biofilm efficiency of 10 typical D-amino acids determined by crystal violet staining. Red stars indicated the five D-amino acids used for subsequent dataset construction.

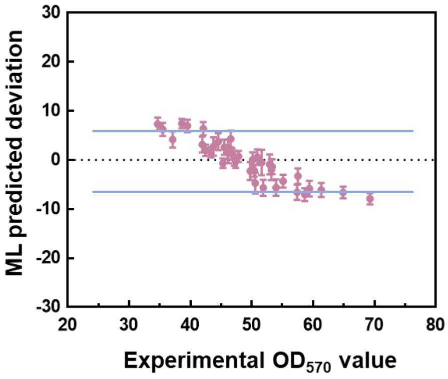

Supplementary Figure 3. The distribution of deviation values between experimental and machine learning predicted values.

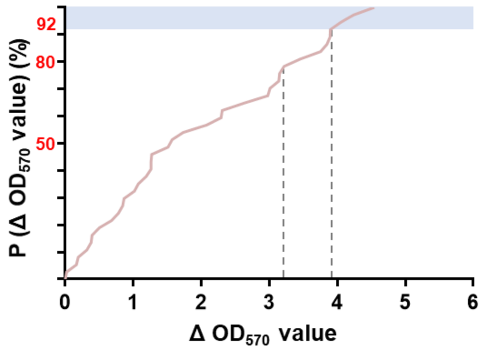

Supplementary Figure 4. The CDF versus prediction error constructed for the refined RF model. The horizontal axis represents the absolute OD_{570} error between experimental and predicted values (ΔOD_{570} value), and the vertical axis represents the proportion of D-amino acids P (ΔOD_{570} value) with errors less than ΔOD_{570} value.

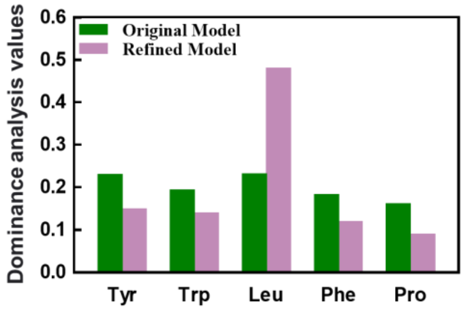

Supplementary Figure 5. Relative importance analysis of the five D-amino acids in the original model and the EGO refined model.

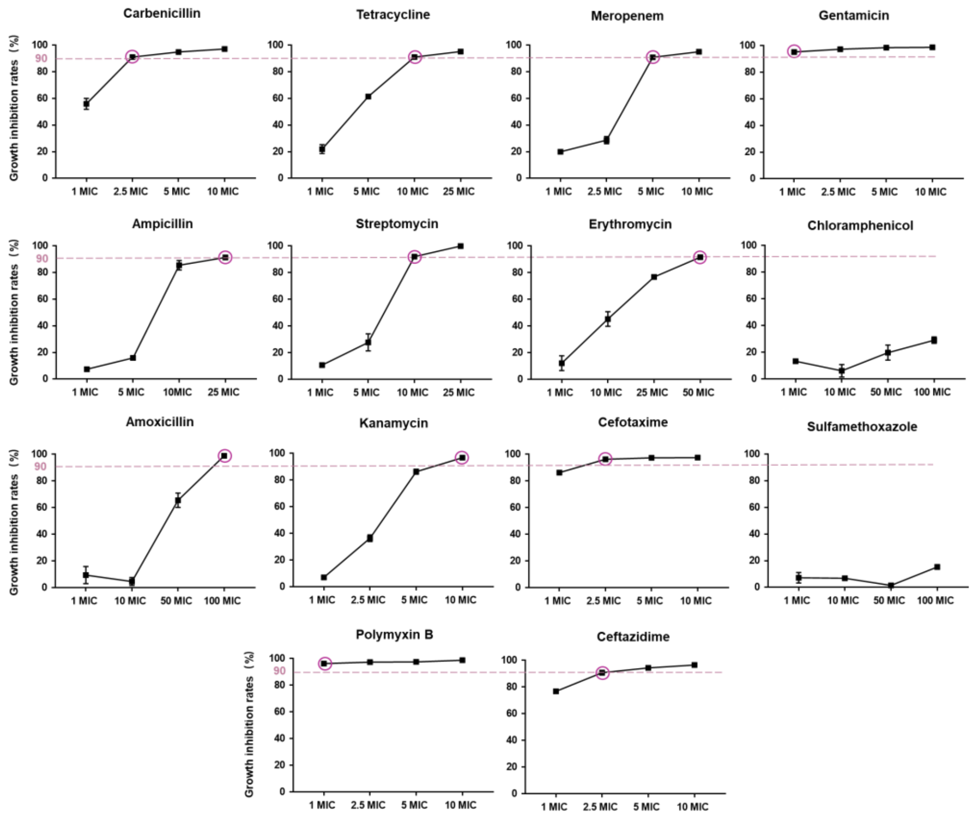

Supplementary Figure 6. High-throughput screening results of the inhibition rates of the antibiotics to *P. aeruginosa* at different MICs. The purple circle represents the LEC of each antibiotic against *P. aeruginosa*.

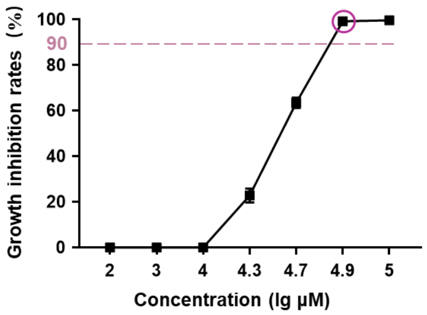

Supplementary Figure 7. The growth inhibition rates of the D-mix to P. aeruginosa at different concentrations.

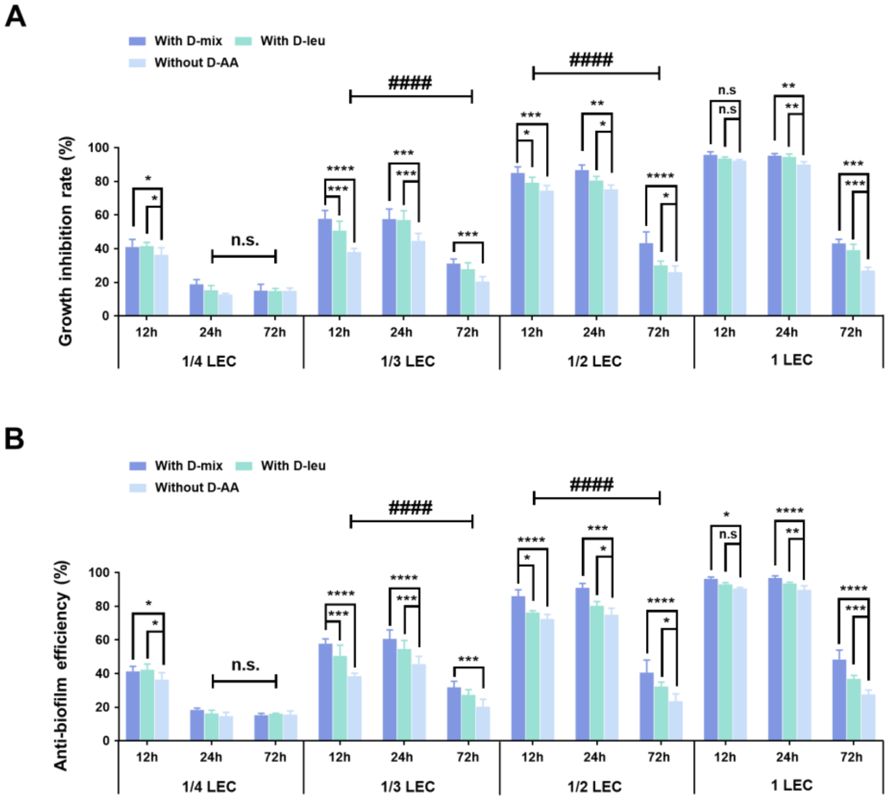

Supplementary Figure 8. The growth inhibition rates of *P. aeruginosa* by the antimicrobial cocktails with different (A) ampicillin and (B) amoxicillin dosages at 12, 24 and 72 hours. Two-way analysis of variance was used for statistical analysis. Data are means ±SD (n = 4; * indicates $p < 0.05$, ** indicates $p < 0.01$, *** indicates $p < 0.001$, and **** indicates $p < 0.0001$). The statistical differences relating to D-mix and

antibiotic combinations with different incubation times are represented by doubles symbols (# indicates $p < 0.05$, ## indicates $p < 0.01$, ### indicates $p < 0.001$, and #### indicates $p < 0.0001$).

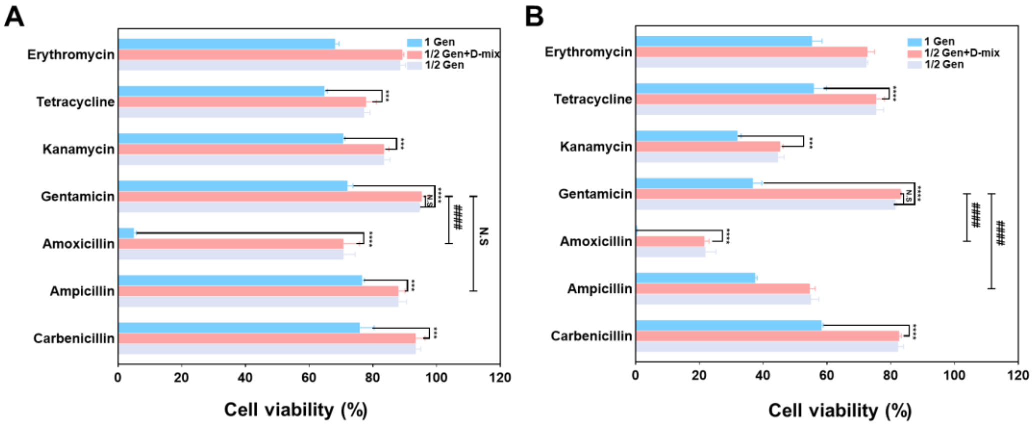

Supplementary Figure 9. The toxicity to MC3T3-E1 cell lines measured by CCK-8 assay after A) 24 hours and B) 72 hours of incubation.

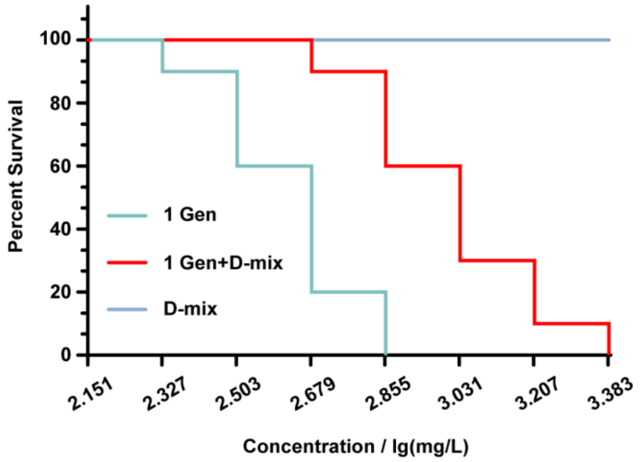

Supplementary Figure 10. The percent survival of mice (n = 6) after injecting different concentrations of drugs at 24 hours.

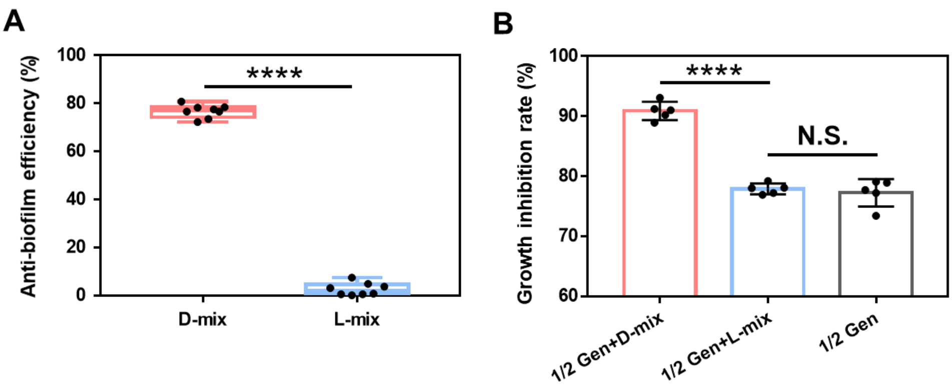

Supplementary Figure 11. (A) Anti-biofilm efficiency of *P. aeruginosa* via D-mix and corresponding L-mix. (B) The growth inhibition rates of *P. aeruginosa* via combination therapy with different amino acids usage at 24 hours.

Supplementary Table 1. The original dataset collection methods. Letters A, B, C, D and E represent D-tyrosine (D-try), D-tryptophan (D-trp), D-leucine (D-leu), D-phenylalanine (D-Phe) and D-proline (D-Pro), respectively. Number 1, 2, 3, 4 and 5 before the letters represents the 20%, 40%, 60%, 80%, 100% molar ratios of each component in D-amino acid mixture, respectively. While measuring each experimental group, a set of control groups will be measured accordingly.

[illegible]1	#2	#3	#4	#5	#6	#7	#8	#9	#10	#11	#1[illegible]
[illegible]A	5B	5C	5D	5E	A+4B	4A+B	2A+3B	3A+2B	A+4D	4A+D	2A[illegible]
[illegible]A+2D	A+4E	4A+E	2A+3E	3A+2E	B+4C	4B+C	2B+3C	3B+2C	B+4D	4B+D	2B[illegible]
[illegible]B+2D	B+4E	4B+E	2B+3E	3B+2E	A+2B+2C	2A+B+2C	2A+2B+C	A+B+3C	3A+B+C	A+3B+C	A+[illegible]
[illegible]A+B+2D	2A+2B+D	A+B+3D	3A+B+D	A+3B+D	A+2B+2E	2A+B+2E	2A+2B+E	A+B+3E	3A+B+E	A+3B+E	A+[illegible]
[illegible]A+C+2D	2A+2C+D	A+C+3D	3A+C+D	A+3C+D	A+2C+2E	2A+C+2E	2A+2C+E	A+C+3E	3A+C+E	A+3C+E	A+[illegible]
[illegible]A+D+2E	2A+2D+E	A+D+3E	3A+D+E	A+3D+E	B+2C+2D	2B+C+2D	2B+2C+D	B+C+3D	3B+C+D	B+3C+D	B+[illegible]
[illegible]B+C+2E	2B+2C+E	B+C+3E	3B+C+E	B+3C+E	B+2D+2E	2B+D+2E	2B+2D+E	B+D+3E	3B+D+E	B+3D+E	A+[illegible]
[illegible]A+B+C+D	A+B+2C+D	A+2B+C+D	A+B+C+2E	2A+B+C+E	A+B+3C+E	A+2B+C+E	A+B+D+2E	2A+B+D+E	A+B+2D+E	A+2B+D+E	A+[illegible]

Supplementary Table 2. Anti-biofilm efficiency of D-amino acids against *P. aeruginosa* reported in the literature.

Strains	D-amino acid types	Concentration (μM)	Efficiency (%)	References
ATCC 15692	Met/Phe/Trp	5,000	70	[1]
ATCC 15692	Phe	5,000	55	[1]
PAO1	Cys	6,000	32	[2]
PAO1	Cys	4,000	30	[2]
PAO1	Cys	2,000	22	[2]
PAO1	Trp/Tyr/Cys	6,000	29	[2]
PAO1	Trp/Tyr/Cys	4,000	28	[2]
PAO1	Trp/Tyr/Cys	2,000	23	[2]

PAO1	Trp/Tyr/Cys	1,000	14	[2]
PAO1	His/Thr/Trp/Ser	6,000	55	[2]
PAO1	his/thr/trp/Ser	4,000	42	[2]
PAO1	His/Hhr/Hrp/Ser	2,000	24	[2]
PAO1	His/Hhr/Hrp/Ser	1,000	12	[2]
ATCC 700829	Leu	152	60	[3]
ATCC 15692	Trp	5,000	50	[4]
ATCC 15692	Trp	10,000	56	[4]
ATCC 700829	Tyr	20	13	[5]
ATCC 700829	Tyr	50	22	[5]
ATCC 700829	Tyr	100	28	[5]
ATCC 700829	Tyr	5	15	[5]
ATCC 700829	Tyr	0.5	16	[5]

Supplementary Table 3. MIC values of antibiotics obtained from the EUCAST (https://www.eucast.org/) database and other references.

Antibiotic	MIC (mg L^{-1})	Reference
Gentamicin	8	Eucast
Carbenicillin	64	[6]
Tetracycline	16	Eucast
Cefotaxime	10	Eucast
Ceftazidime	2	Eucast
Chloramphenicol	4	[7]
Erythromycin	4	[8]
Amoxicillin	64	[9]
Ampicillin	64	[9]
Kanamycin sulfate	64	Eucast
Streptomycin	5	[10]
Polymyxin B	4	[11]
Meropenem	0.5	Eucast
Sulfamethoxazole	4	[12]

Supplementary Table 4. The category and LECs of antibiotics in this work.

Category	Antimicrobial agent	LEC (mg/L)
**β-lactamase inhibitor**	**Carbenicillin**	**160**
	**Ampicillin**	**1600**
	**Amoxicillin**	**6400**
	**Meropenem**	**2.5**
	**Chloramphenicol**	**NS**
	**Cefotaxime**	**25**
	**Ceftazidime**	**5**
**Aminoglycoside**	**Gentamicin**	**8**
	**Kanamycin**	**640**
	**Streptomycin**	**50**
**Tetracyclines**	**Tetracycline**	**160**
**Peptides**	**Polymyxin B**	**4**
**Sulfonamides**	**Sulfamethoxazole**	**NS**
**Macrolide**	**Erythromycin**	**200**

Supplementary Table 5. Descriptor values of some β-lactamase antibiotics.

Antibiotics	Fraction CSP3	Chi0	PMI3	RadiusOf Gyration	HBA	HallKier Alpha	Spherocity Index	Labte
**ampicillin**	**0.438**	**17.64**	**4058**	**3.5**	**7**	**-2.06**	**0.27**	**143**
**amoxicillin**	**0.438**	**18.52**	**5496**	**3.9**	**8**	**-2.26**	**0.30**	**147**
**cefotaxime**	**0.375**	**22.00**	**10099**	**4.8**	**12**	**-3.13**	**0.13**	**177**
**ceftazidime**	**0.318**	**26.90**	**13359**	**5.1**	**13**	**-3.98**	**0.20**	**218**

Experimental Section

In vivo toxicity test: The C57 mice were randomly divided into 3 groups (10 per group). Each of the mice was injected intravenously with drugs or drug combinations at different doses (i.e.,142, 213, 320, 480, 720, 1080, 1620, 2430 mg/kg, 0.2 mL/20 g). LD_{50} values were estimated from the survival curve of treated mice over 1 day using the Spearman-Karber method. The kidney and liver functions were also assessed *in vivo*. The mice were randomly divided into 4 groups: PBS control group, 1 Gen, 1/2 Gen +

D-mix and D-mix treated groups (6 mice per group). Each mouse received injections of 64 mg/mL for 1 Gen groups, 32 mg/mL gentamicin coupled with 800 μm/mL D-mix for 1/2 Gen + D-mix groups, or 800 μm/mL D-mix for D-mix groups. The mice were then euthanized to obtain blood samples from their periorbital plexus at 24 hours post infection for analysis of kidney (creatinine, urea nitrogen) and liver functions (alanine transaminase-ALT, aspartate transaminase-AST).

Hemolysis assay: The hemolytic activities of the drugs were tested via fresh rat red blood cells (rRBCs). Firstly, rRBCs were diluted to achieve 4% v/v of blood content in PBS. The drugs were dissolved in PBS at concentrations of 64 mg/mL for 1 Gen groups, 32 mg/mL gentamicin coupled with 800 μm/mL D-mix for 1/2 Gen + D-mix groups and 800 μm/mL D-mix for D-mix groups. 100 μL volumes of drug solutions were then mixed with equal volumes of diluted blood suspension. The mixtures were incubated at 37 °C for full interactions between rRBC and drug solution. Then, the mixture was centrifuged for 5 min at 4 °C. After that, 100 μL supernatant was dispensed into a 96-well microplate. A microplate reader was used to measure the absorbance of samples at 576 nm. Untreated rRBC suspension was set as the negative control and rRBC suspension treated with 0.1% Triton-X was set as the positive control. Each sample was measured in triplicates. The hemolytic activities were calculated by the following equation (1):

$$\text{Hemolytic activities} = (T_{OD}-N_{OD})/(P_{OD}-E_{OD}) \times 100\% \qquad (1)$$

in which T_{OD}, N_{OD} and P_{OD} are the OD_{576} values of the treated samples, the negative control and the positive control, respectively.

[1] S. Wang, X. Sun, W. Gao, Y. Wang, B. Jiang, M. Afzal, C. Song, S. Wang *Colloids Surf. B*, **2018**, *164*, 20.

[2] P. She, L. Chen, H. Liu, Y. Zou, Z. Luo, A. Koronfel, Y. Wu *Microb. Pathog*. **2015**, *86*, 38.

[3] C. Rumbo, J. A. Vallejo, M. P. Cabral, M. Martínez-Guitián, A. Pérez, A. Beceiro, G. Bou *J. Antimicrob. Chemother*. **2016**, *71*, 3473.

[4] C. J. Sanchez, K. Akers, D. Romano, R. Woodbury, S. Hardy, C. Murray, J. Wenke *Antimicrob. Agents Chemother*. **2014**, *58*, 4353.

[5] C. Yu, X. Li, N. Zhang, D. Wen, C. Liu, Q. Li, *Water Res*. **2016**, *92*, 173.

[6] D. Kwon, C. Lu *Antimicrob. Agents Chemother*. **2006**, *50*, 1623.

[7] Y. Morita, J. Tomida, Y. Kawamura *Front. Microbiol*. **2014**, *4*, 422.

[8] K. W. Tsang, P. Ng, P. L. Ho, S. Chan, G. Tipoe, R. Leung, J. Sun, J. C. Ho, M. S. Ip. W. K. Lam *Eur. Respir. J*. **2003**, *21*, 401.

[9] H. H. Handsfield, H. Clark, J. F. Wallace, K. K. Holmes, M. Turck *Antimicrob. Agents Chemother*. **1973**, *3*, 262.

[10] J. T. Tseng, L. E. Bryan, H. M. V. D. Elzen *Antimicrob. Agents Chemother*. **1972**, *2*, 136.

[11] M. Berditsch, T. Jäger, N. Strempel, T. Schwartz, J. Overhage, A. S. Ulrich *Antimicrob. Agents Chemother*. **2015**, *59*, 5288.

[12] P. Huovinen *Clin. Infect. Dis*. **2001**, *32*, 1608.
